# Hydrogel with silver nanoparticles synthesized by *Mimosa tenuiflora* for second-degree burns treatment

**DOI:** 10.1038/s41598-021-90763-w

**Published:** 2021-05-28

**Authors:** Aaron Martínez-Higuera, César Rodríguez-Beas, Jesús Mauro Adolfo Villalobos-Noriega, Abraham Arizmendi-Grijalva, Carlos Ochoa-Sánchez, Eduardo Larios-Rodríguez, Juan Manuel Martínez-Soto, Ericka Rodríguez-León, Cristina Ibarra-Zazueta, Roberto Mora-Monroy, Hugo Alejandro Borbón-Nuñez, Alfonso García-Galaz, María del Carmen Candia-Plata, Luis Fernando López-Soto, Ramón Iñiguez-Palomares

**Affiliations:** 1 Department of Physics, Nanotechnology Graduate Program, University of Sonora, Rosales and Transversal, 83000 Hermosillo, Sonora Mexico; 2 Department of Chemical and Metallurgical Engineering, University of Sonora, Rosales and Transversal, 83000 Hermosillo, Sonora Mexico; 3 Department of Medicine and Health Science, University of Sonora, Rosales and Transversal, 83000 Hermosillo, Sonora Mexico; 4 Department of Agriculture and Livestock, University of Sonora, Road to Kino Bay km 20.5, Hermosillo, Sonora Mexico; 5 Department of Physic Researching, University of Sonora, Rosales and Transversal, 83000 Hermosillo, Sonora Mexico; 6 CONACYT-Centro de Nanociencias Y Nanotecnología, UNAM, Km 107 Carretera Tijuana-Ensenada s/n, 22800 Ensenada, B.C. C.P Mexico; 7 Food Science Coordination, Research Center in Food & Development (CIAD), Road Gustavo Enrique Astiazarán Rosas, No. 46, Col. La Victoria, 83304 Hermosillo, Sonora Mexico

**Keywords:** Drug discovery, Materials science, Nanoscience and technology

## Abstract

In this work we use *Mimosa tenuiflora* (MtE) extracts as reducing agents to synthesize silver nanoparticles (AgMt NPs) which were characterized by DPPH and Total Polyphenols Assays, UV–visible, X-ray diffractometer (XRD), high-resolution transmission electron microscopy (HRTEM), X-ray photoelectron spectroscopy (XPS) and Thermogravimetric analysis (TGA). AgMt NPs possess average sizes of 21 nm and fcc crystalline structure, it was also confirmed that the MtE is present in the AgMt NPs even after the cleaning protocol applied. Subsequently, carbopol hydrogels were made and the MtE and the synthesized AgMt NPs were dispersed in different gels (MtE-G and AgMt NPs-G, respectively) at 100 µg/g concentration. The gels were characterized by UV–Vis, IR, and rheology. Antimicrobial tests were performed using *Staphylococcus aureus* and *Escherichia coli*. Burn wound healing was evaluated in a second-degree burn injury on a Wistar rats model for 14 days and additional skin biopsies were examined with histopathological analysis. Gel with commercial silver nanoparticles (Ag NPs) was prepared and employed as a control on the biological assays. Hydrogel system containing silver nanoparticles synthesized with *Mimosa tenuiflora* (AgMt NPs-G) is a promising therapeutic strategy for burn wound healing, this due to bactericidal and anti-inflammatory effects, which promotes a more effective recovery (in percentage terms) by damaged area.

## Introduction

Thermal burns occur when skin cells are destroyed by hot liquids, hot solid objects or direct exposure to fire^1^. Burn injuries are a global health problem accentuated in poor countries and those injuries are associated with high costs in public health systems, this due to prolonged hospitalization periods and rehabilitation with possible infectious processes that can lead to death from sepsis. According to the World Health Organization (WHO), in 2017 there were approximately 180,000 burn-related deaths where 2/3 parts focused on Africa and South-East Asia. In India almost 1 million people suffer moderate or severe burns annually. In Mexico, according to INEGI, an average of 128,000 cases of burns occurs where 1/3 of them are minors, being the most frequent injuries in the home.

Fatemi et al^2,3^ compared the effects of green tea extracts and sulfadiazine in second degree burns induced in rats. They found that the topical treatment with green tea extracts is an effective alternative in second degree burns healing. The authors point out that the polyphenolic compound EGCG is the molecule responsible for the healing process improving.

Several plants have been used for compounds extraction with bioactivity, specifically to be evaluated as healing agents, among them we can find *Mimosa tenuiflora*. This plant has compounds such as alkaloids, chalcones, steroids, polyphenols, polysaccharides and terpenoids, which are known to promote fibroblast division and promote signaling pathways aimed at inhibiting inflammation and pain^4–6^. It is also known that *M. tenuiflora* extract has a powerful bactericidal and fungicidal effect. However, there is not enough information on how *M. tenuiflora* extract exerts its bioactivity.

There is little information on metallic nanomaterials synthesis using *M. tenuiflora* extracts, same which we believe can potentiate extract bioactive effect on the hydrogel or colloidal nanoparticle presentation. In recent years, efforts have been made to develop hydrated and biocompatible composite materials that have broad spectrum antimicrobial activity for topical applications focused on burns treatment and chronic injuries. In this context, several proposals of different hydrogel matrices based on acrylic acid (carbopol)^7–10^, catechol^11^, natural polysaccharides (starch, chitosan, alginate)^12–15^, or short chain peptides^16–18^, have proved to be a good option as biocompatible support materials for microbicidal agents dispersion. The search for new antimicrobials has made metal nanoparticles an emerging alternative on infections treatment associated with difficult-to-recover lesions such as varicose ulcers, diabetic foot^19,20^ or skin burns. Silver and gold nanoparticles colloidal solutions have shown a broad-spectrum microbicidal effect including *Staphylococcus aureus* and *Pseudomonas aeruginosa*, two multi-resistant pathogens that have persistent presence in burn injuries^21,22^.

Silver nanoparticles have antibacterial effects. Various authors discuss their mechanism of action: (a) the interaction between Ag NPs and the bacterial membrane cause rupture that conduce to microorganisms' death. Gram-negative bacteria show more susceptibility than Gram-positive bacteria because the thickness wall is around ten times less for Gram-negative^23–25^. (b) The silver ions Ag^+^ interact with negatively charged wall cellular bacteria forming pores over the membrane that frees DNA and proteins, which leads to death cell^26,27^. (c) The AgNPs are donators of electrons to the molecular oxygen that result in the formation of radical reactive superoxide (O_2_^−^)^28,29^; this leads to AgNPs generate reactive oxygen species (ROS) that promote interruption of ATP production and DNA replication^24,26^. (d) AgNPs reaction with sulfur groups (proteins), particularly with thiols groups (–SH)^23,30^ and phosphates (DNA), generating failures in the replication mechanism of the bacteria^25^. (e) Other proposals include the surface area that serves as transport mechanisms for antibiotics or bioactive molecules that generate antibacterial action by depositing on the cell membrane. Nanoparticles' size is an essential parameter because the smallest nanoparticles and larger surface area promote the most antibacterial activity^29,30^. At this point, it can modulate other parameters to obtain biocompatibility as surface area, concentration, colloidal state, surface functionalization, and surface charge. All are parameters for enhance cytotoxic properties of AgNPs and generate synergy antibacterial using AgNPs and their biomolecules surface.

In this work we propose to use hydro-ethanol solvents to obtain *Mimosa tenuiflora* extracts to be use as bio-reducing agents on metal nanoparticles synthesis (Ag). Subsequently, a carbopol-based hydrogel will be formulated as a matrix to disperse the *M. tenuiflora* extract and Ag nanoparticles. The final product will be a composite material that improves burn injuries healing and inhibits infections development of the treated area.

## Materials and methods

### Nanoparticles synthesis

Fresh bark of *Mimosa tenuiflora* was collected in Cintalapa, Chiapas, México. The bark was washed several times to remove dust and dried at 50 $^\circ{\rm C}$ in a convection oven (Ecoshel, model 9023A) for 1 week. *Mimosa tenuiflora* tree bark was cut in small pieces and 15 g were deposited in a 100 mL flask, which contained a mixture of ethanol (Fermont, 99% purity) and ultra-pure water in a 70:30 proportion. The flask was kept in dark at room temperature. After 15 days, the obtained MtE was filtered, rotoevaporated and lyophilized. For AgMt NPs synthesis, an MtE aqueous solution was prepared with MtE powder at 38.88 g/L. As metal precursor AgNO_3_ 0.1 M aqueous solution was used. Then, in a 100 mL flask, 65.6 mL of ultrapure water, 7.2 mL Mt extract, and 7.2 mL AgNO_3_ were added and mixed under magnetic stirring (750 rpm) for one hour at room temperature. Once nanomaterial was synthesized, it was centrifuged at 14,000 rpm for one hour and supernatant was discarded. The precipitated solids were resuspended in water and sonication was used for 15 min to redisperse them. The centrifugation-redispersion cycle was repeated two times. Finally, precipitated material was resuspended in ultrapure water and redispersed for further assays. Concentration was estimated by atomic absorption.

Commercial silver nanoparticles (Ag NPs) synthesized by chemical methods and stabilized with polyvinylpyrrolidone (PVP) were acquired from Sigma-Aldrich as a control for antibacterial and burn healing assays. The commercial nanoparticles were chosen with morphology and size similar to those produced in our synthesis with *Mimosa tenuiflora* extracts.

### Hydrogel formulation

Hydrogels were produced using glycerol (2.75 g), carbopol-940 (0.825 g), DMSO (1.2 g), and triethanolamine. All reagents were acquired in Sigma-Aldrich. Once hydrogel base compounds were mixed AgMt NPs (100 µg/g), Ag NPs (100 µg/g) and MtE (100 µg/g) were added separately to hydrogels, and the final mix was taken to a weight of 110 g using sterile water. All formulations were stirred under magnetic stirring at 700 rpm for 12 h at room temperature. Immediately afterward, triethanolamine was added dropwise maintaining vigorous stirring until reaching the gel consistency and pH ~ 6.3. As a negative control the base formulation was used.

### DPPH and total polyphenols assay

For DPPH (2,2-diphenyl-1-picrylhydrazyl) assays, all tests were done by triplicate. Different Mt extract concentrations (25, 12.5, 6.25, and 3.125 μg/mL) were tested. One hundred microliters of ethanol were added to 100 μL of each concentration, in addition to the DPPH solution (300 μM). Samples were incubated for 2 h in the dark before measuring absorbance at 517 nm. The obtained results were compared with Trolox (70 μmol/L). For scavenging activity, DPPH radical was dissolved in ethanol and used as a blank^31,32^.

Scavenging activity percentage was calculated with Eq. (1) where A sample is the sample absorbance and A control is the blank absorbance. Data were analyzed using variance analysis (ANOVA) with Tukey multiple comparison tests.

1 $$\% Scavenging \, activity \, = \, \left[ {\left( {1 \, - \, A \, sample} \right) \, /A \, control} \right] \times 100.$$

For total polyphenol assay, same concentrations were used by adding Folin Ciocalteu at 0.25 N and sodium carbonate at 5% with a 1-h incubation in light absence. Absorbance was measured at 750 nm, on a microplate reader Multiskan FC, Thermo Scientific. The obtained results are expressed as gallic acid equivalents^33,34^.

### UV–Vis spectra

A double-beam Perkin-Elmer Lambda 40 UV–Vis spectrometer was used in the characterization of all systems. Espectra were captured at a speed of 240 nm/min and a slit of 0.5 nm was selected. For MtE and AgMt NPs, experiments were carried out in quartz cells using water as solvent, and 3 µL and 100 µL of stock samples were added, respectively. Spectra were collected from 220–900 nm. For gel systems (G, MtE-G and AgMt NPs-G), samples were deposited on slides. The reading was made by placing the slide vertically and securing the position to ensure normal incidence of the light beam. A clean slide was used as reference. Spectra were collected from 325 to 900 nm.

### TEM and HR-TEM

Nanoparticle’s morphology and size distribution were analyzed by transmission electron microscopy (Field Emission JEOL 2010 operated at 200 keV), 10 μL of the sample were deposited on copper grids covered with a fomvar-carbon film (Electron Microscopy Sciences, 300 Mesh). Grids left to dry for 1 h and placed in a vacuum chamber for 12 h. Interplanar spacings of crystal planes revealed by high resolution TEM (HRTEM) were determined using Digital Micrograph software^35^ (Version 3.7, Gatan, Inc. https://www.gatan.com/products/tem-analysis/gatan-microscopy-suite-software).

### XRD

Powder X-ray diffractograms were collected at 300 K using a Rigaku XtalLAB SuperNova System, Cu Kα radiation (λ = 1.5417 Å) source, equipped with a HyPix3000 X-ray detector in transmission mode operating at 50 kV and 1 mA. Powder samples were mounted in MiTeGen microloops. Powder diffractograms were collected over an angular 2θ range between 35° and 90° with a step size^36^ of 0.01°.

### XPS

X-ray photoelectron spectroscopy assays were carried out on a Perkin Elmer model PHI 5100, which contains a dual source of Mg/Al, 300 W, 15 kV. The Al Kα emission line with energy of 1486.6 eV was used for MtE XPS characterization. For AgMt NPs the Mg Kα emission line (1253.6 eV) was employed. Both experiments were performed under vacuum conditions of 2 × 10^–9^ Torr. Ag 3d5/2 peak at 368.3 eV was used to adjust AgMt NPs spectrum and the C 1 s line at 284.6 eV was used for MtE spectrum. Data was analyzed using Spectral Data Processor (SDP) software.

### Zeta potential and DLS

AgMt NPs and Ag NPs zeta potentials (ζ) were measured with Zetasizer NS (Malvern, PA). Also, sizes were measured by dynamic light scattering (DLS) by Zetasizer NS (resolution of 0.5 nm). The samples were placed into a U-shaped folded capillary cell for ζ measurements. Each sample was measured at room temperature (25 °C) in triplicate.

### TGA

MtE and AgMt NPs thermogravimetric analysis (TGA) were conducted using a Perkin Elmer Pyris 1 at a constant heating rate of 10 °C min^−1^ from 25 to 800 °C under nitrogen atmosphere.

### FTIR

Samples were analyzed through FTIR Perkin-Elmer Frontier provided by an GladiATR diamond accessory. The spectrum was obtained on transmittance mode and was scanned registering the spectrum with 32 scans at a resolution of 2 cm^−1^, from 4500 to 500 cm^−1^_._ MtE and AgMt Nps was measured in dust and G, MtE-G, AgMt NPs-G, Ag NPs-G and Ag NPs in liquid.

### Rheometry

Measurements were carried out on a MCR502 (Anton Paar, Ostfildern, Germany) of parallel plates with 1 mm of separation and number plate PP50 SN43778. Test was performed at 37 °C. Analyzed systems were G, MtE-G and AgMt-G.

### Inoculum preparation and gel antimicrobial assay

The stock cultures were maintained in brain heart infusion (BHI, BD), added with 20% glycerol at − 20 °C. Both cultures, *S. aureus* (ATCC 6538P) and *E. coli* (ATCC 25,922) were inoculated into BHI and incubated 24 h at 36 ± 1 °C for reactivation purpose. After that, the cultures were inoculated into BHI agar for purity confirmation. Once purity was confirmed, cultures were inoculated into Müeller Hinton Broth, incubated overnight at 36 ± 1 °C. Cultures were adjusted using McFarland nephelometer 0.5 tube and massive inoculated into Müeller Hinton Petri dishes. After massive inoculum was absorbed by agar, 100 mg of each gel (G, MtE-G, Ag NPs-G, and AgMt NPs-G) was poured over and incubated as previously described.

### Minimal inhibitory concentration (MIC) and minimal bactericidal concentration (MBC) of silver nanoparticles and extract

Both *S. aureus* and *E. coli* were inoculated each one into Müeller Hinton broth and adjusted to 0.5 McFarland Nephelometer as previously described. Once inoculums were adjusted for MIC determination microdilution test was performed. Briefly, a 96 well plate was used for this purpose. 180 µL of fresh Müeller Hinton were poured into 5 wells, each well was added with 10 µL of adjusted inoculum and 10 µL of each solution. Tested agents were AgMt NPs, Ag NPs, MtE and ultra-pure water as vehicle in 1:2 serial dilutions from 100 to 6,25 µg/mL. Six extra wells were poured with Müeller Hinton broth, 3 of them were added with same inoculum and these wells were used as negative and positive control, respectively. After that, 96 well plate was incubated overnight at 36 ± 1 °C. After incubation, for MIC determination each clear well was considered as inhibited culture and each turbid well as negative for inhibition. The concentration of tested solution with inhibited culture was recorded as MIC value.

For MBC determination each well of MIC determination were reinoculated into Müeller Hinton agar and incubated overnight under same previously described conditions. Each negative culture (with absence of visible colonies) was considered as bactericidal effect and respectively solution concentration was recorded as MBC value.

### Cytotoxic assays

MtE and AgMt NPs cytotoxic effect was evaluated in HUVEC cells (Gibco, Cat. #: C0035C) using calcein (Thermofisher) as a cellular viability indicator. HUVEC cells were grown in DMEM medium (Sigma-Aldrich), supplemented with FBS 10% (Gibco). HUVEC cells were grown at 37 °C at 5% CO_2_ and once cells reached confluence, they were harvested using trypsin (Sigma-Aldrich). Cells were counted and adjusted at 5 × 10^5^ cells per-ml to be seeded in a 24 multiwell plate (Costar). Cells were treated with control without stimulus, MtE and AgMt NPs with concentrations 12.5, 25, 50, 100 μg/mL.

Treatments were allowed to interact with cells for 24 h. Once time was over, HUVEC cells were harvested and resuspended in PBS 1X. For calcein staining, 2 µL of a 50 µm stock solution were added and then incubated in dark for 15 min. Viable cells percentage was analyzed by flow cytometry. All treatments were done by triplicate and for each, 10,000 cells were counted on a BD FACS VERSE (Becton Dickinson). Data analyses were performed with BD FACSuite (BD Biosciencess).

### Burn healing assays

15 healthy male Wistar rats (3 to 4 months old) with an average weight of 200 to 250 g were used to induce second degree burn injuries. All rats were kept in 12 h of light/dark at a controlled temperature of 25 °C. Water and food were provided ad libitum. The experimental protocol was approved by the University of Sonora Bioethics Committee (DMCS/CBIDMCS/D-125), also the Mexican standard for management and use of animals was followed (NOM-033-ZOO-1995), finally, all the procedures related to the care of the experimental animals and experimental procedures were based on Sect. 8a and 9 respectively of Essential 10 of the ARRIVE guidelines. Rats were anesthetized with Xilazine/Ketamine (8 mg/kg + 70 mg/kg) by intraperitoneal administration. Fur was trimmed and shaved. For injury induction, a soldering iron model WLC100 with adjustable temperature was acquired (Weller). The soldering iron tip was removed and replaced with a 17 mm perimeter cylindrical metal head. Modified soldering iron was ignited and adjusted to 110 °C to contact with skin for 10 s. Skin burn injury was performed on the side of rats 3 cm from spine. A tissue sample was taken to establish damage on day 0. Experimental groups models consisted of untreated rats (negative control), rats treated with hydrogel base formulation (G, vehicle control) rats treated with MtE-G (100 µg/g), rats treated with AgMt NPs-G (100 µg/g) and rats treated with Ag NPs-G (100 µg/g). All groups were administered 500 mg of hydrogel except for negative control. Wound recovery process was measured with an electronic Vernier (YKS). After every applied treatment, a patch and bandage were placed on the treated area. After experiment, a biopsy was taken and fixed with formalin free tissue fixative (Sigma) for histopathological analysis.

### Statistical analyses

Samples were compared using a one-way analysis of variance. In all cases, post hoc comparisons of individual groups' means were performed using Tukey's significant difference test, where p-value < 0.05 denoted significance. This statistical analyzed were performed using the software IBM SPSS Statistics Base 22 (IBM Corp. Released 2013. IBM SPSS Statistics for Windows, Version 22.0. Armonk, NY: IBM Corp. https://www.ibm.com/analytics/spss-statistics-software). All statistical analyzed were based on Sect. 7a of Essential 10 of the ARRIVE guidelines.

## Results and discussions

### DPPH and total polyphenols assay

MtE total phenolic content was 486 ± 17.47 mg of gallic acid equivalents per gram of extract. This content is slightly higher than that previously reported by our group for *Mimosa tenuiflora* bark extract obtained from another location^35^. Differences could be due to variability in the phytoconstituents relative abundance according to the geographical region where samples were obtained. DPPH assay shows that MtE and AgMt NPs IC_50_ value​​ of 10.04 µg/mL and 18.27 µg/mL, respectively. Bharati et al. report silver nanoparticles biosynthesis using *Diospyros montana* stem bark extract, where they observed an IC_50_ value of 40 µg/mL for their silver nanoparticles^37^. This value represents almost half of the antioxidant capacity obtained for our AgMt NPs. In the same way, AgMt NPs antioxidant capacity is almost half of the MtE, which suggests a high content of the complexed extract in nanoparticles that contributes to the reduction of free radicals in DPPH test^38^. Phenolic compounds high content in MtE and its prominent antioxidant capacity suggest an important reducing potential of this natural agent for metallic NPs synthesis.

### UV–Vis spectra

**Figure 1:**
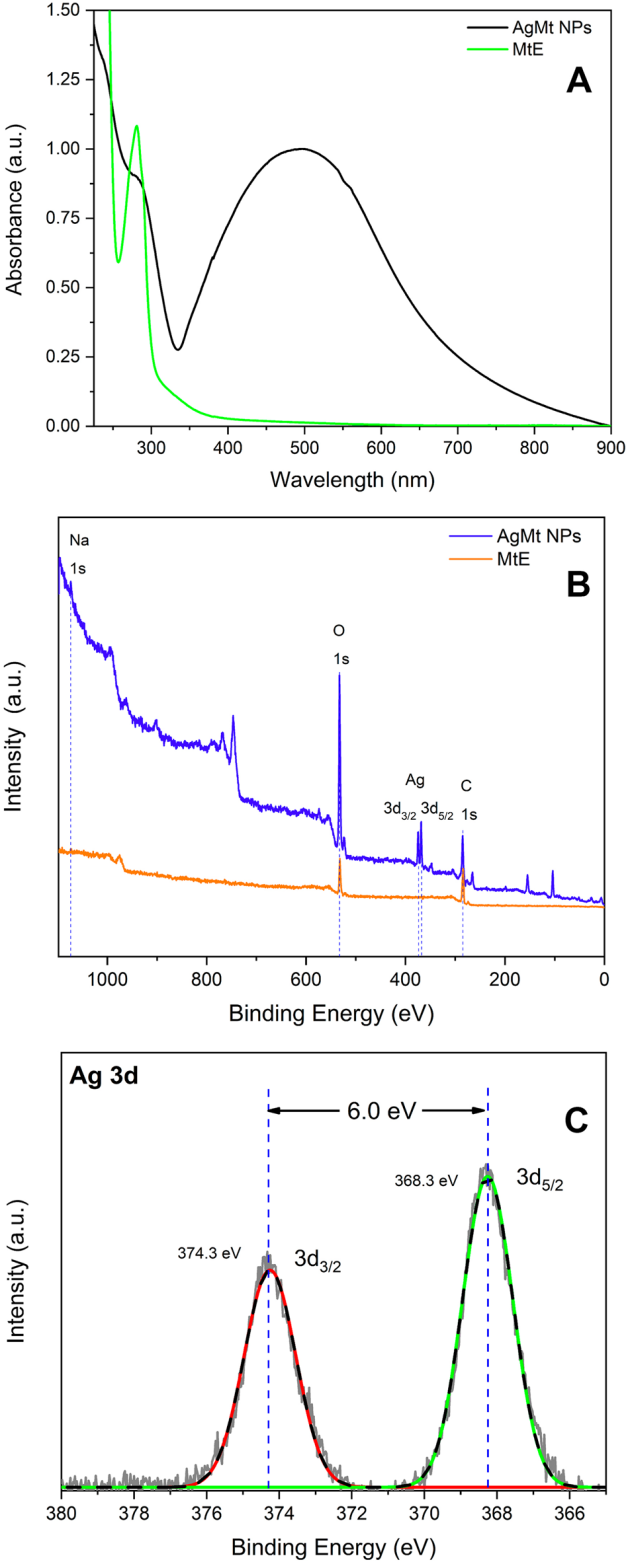
(**A**) UV–Vis absorption spectra of MtE and AgMt NPs. X-ray photoelectron (XPS) spectra (**B**) Complete scan of AgNPS and MtE, (**C**) high-resolution of Ag 3d 3/2 and 3d 5/2.

In Fig. 1A, MtE UV–Vis spectrum consists of a narrow band centered at 280 nm which can be associated with phenolic molecules present in the extract. AgMt NPs UV–Vis spectrum consists of a broadband (FWHM ~ 250 nm) with a maximum at 490 nm, a shoulder can also be observed in the region of 280 nm that indicates the presence of phenolic molecules in nanoparticles. AgMt NPs UV–Vis spectrum analysis in the region of 245 to 325 nm, using the software Origin(Pro), Version 2018 (OriginLab Corporation, Northampton, MA, USA, https://www.originlab.com/ ), allows obtaining MtE contribution to referred spectrum. Using a calibration curve for MtE content, it was possible to estimate MtE concentration in the nanoparticles of 494 µg/mL.

### XPS

MtE survey spectrum (Fig. 1B) consists of two intense peaks located in the regions corresponding to O (~ 532.8 eV) and C (~ 284.8 eV). These same signals are present in AgMt NPs survey spectrum in addition to the characteristic signal for Ag in the 3d region (~ 368 eV). This represents evidence of the presence of compounds from the extract in the final nanometric product. Atomic percentages obtained from AgMt NPs survey spectra for Ag, C, O, and Na are 2.8%, 56.1%, 38.8%, and 2.3% respectively. The under-quantification of silver can be explained by considering that XPS is a surface analysis technique (~ 10 nm deep). Thus, for our system, silver is expected to be detected in a lower amount due to the MtE molecules attached to the surface of AgMt NPs^39^.

High-resolution XPS spectra for Ag NPs is shows in Fig. 1C. Experimental data are adjusted by Gaussian functions and two well defined peaks centered at 368.3 eV and 374.3 eV are obtained. These peaks are associated to Ag spin-orbital components 3d5/2 and Ag 3d3/2, respectively. Energy separation of Ag3d peaks ( $\Delta E=6.0eV$) correspond to metallic silver^40^ Ag^0^.

### TEM and HR-TEM

**Figure 2:**
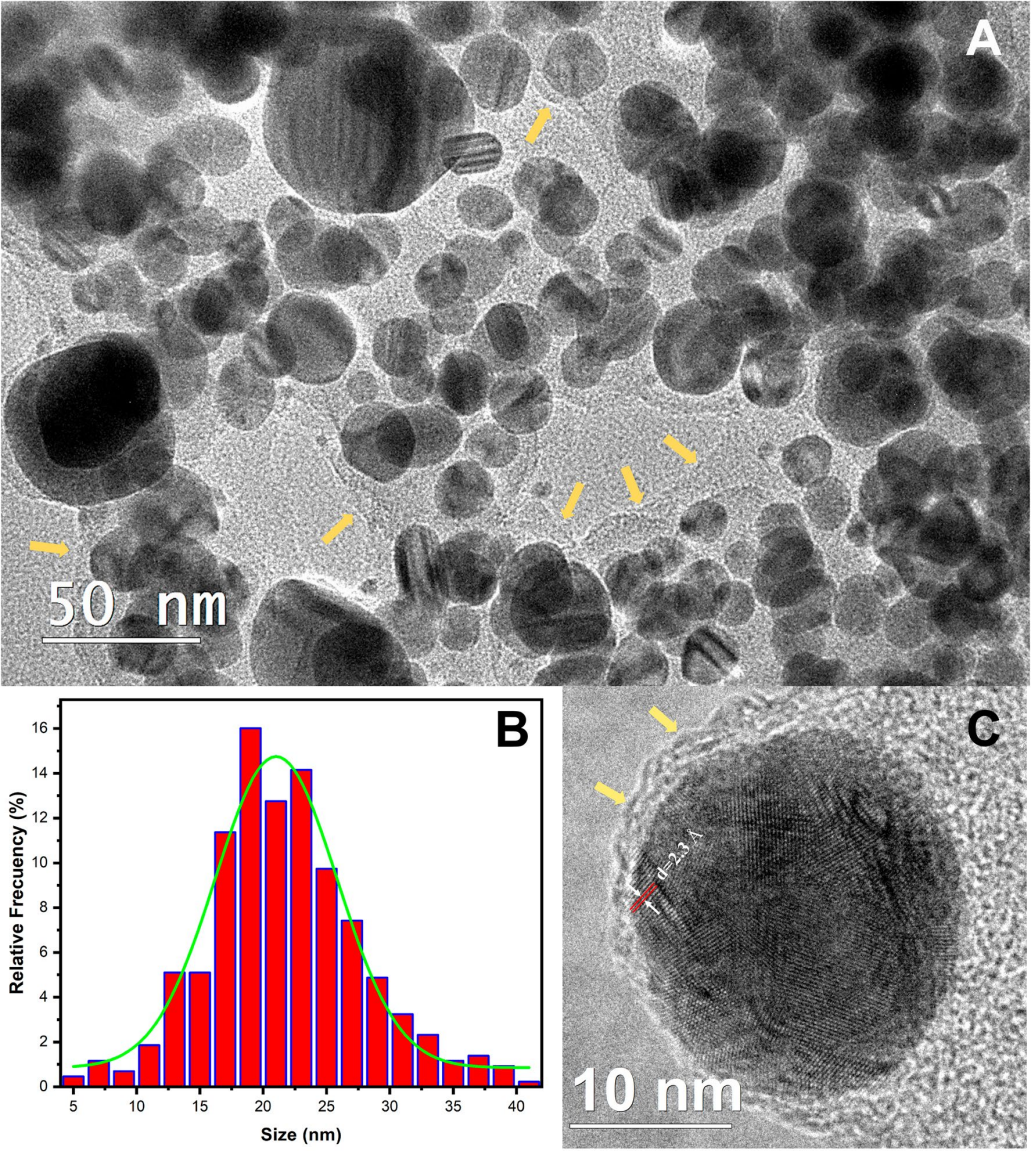
(**A**) TEM micrograph, (**B**) Size distribution histogram with Gaussian fitting, and (**C**) HRTEM micrograph of AgMt NPs.

TEM characterization indicates that AgMt NPs have a quasi-spherical morphology, and some elongated structures are present as shown in the micrograph in Fig. 2A. In the same image, some regions limits are shown with yellow arrows that contrasts markedly from the background and what we consider corresponding to nanoparticles complexed extract. Rodríguez et al. synthesized gold nanoparticles using an *Mimosa tenuiflora* extract and reported the presence of extract around nanoparticles when analyzed by TEM^35^. Figure 2B shows size distribution for AgMt NPs obtained from TEM images. The statistical average size is 21.6 ± 6.1 nm, coinciding with the center of Gaussian distribution which is located at 21 nm with a standard deviation of 4.8 nm. To establish size dispersion behavior in this nanoparticle system, the polydispersity index (PDI) was calculated. PDI is defined as PDI = ${\left(\frac{\sigma }{{R}_{Avg}}\right)}^{2}$ where ${R}_{Avg}$ is the nanoparticles average radius and σ is the corresponding standard deviation^41^. A PDI value less than 0.1 indicates a homogeneous population^42^. In our case, for size estimation by TEM, ${R}_{Avg}=10.8 nm,$ and $\sigma =3.05 nm$, then PDI = 0.079 which indicates a relatively homogeneous population.

Figure 2C corresponds to a single nanoparticle HRTEM. The extract (indicated by the yellow arrows) surrounding the nanoparticle can be observed in more detail. Additionally, some crystalline planes are visible in the image. Particularly, a group of them are shown with their corresponding interplanar distance d = 2.3 Å, measured using Digital Micrograph software. This distance is associated with the planes (111) corresponding to fcc structure of crystalline silver^43^.

The characterization of commercial silver nanoparticles (Ag NPs) by scanning electron microscopy (JSM-7800F, JEOL) and energy-dispersive X-ray spectroscopy, EDX (Quantax XFlash-6, Bruker) shows silver and carbon in the same region. The above suggests that commercial nanoparticles are stabilized by a polymeric agent, as the manufacturer indicates (see Supplementary Fig. 1). Ag NPs SEM micrography indicates that nanoparticles have a quasi-spherical geometry similar to those produced with *Mimosa tenuiflora extract* (see Supplementary Fig. 2A).

### XRD

**Figure 3:**
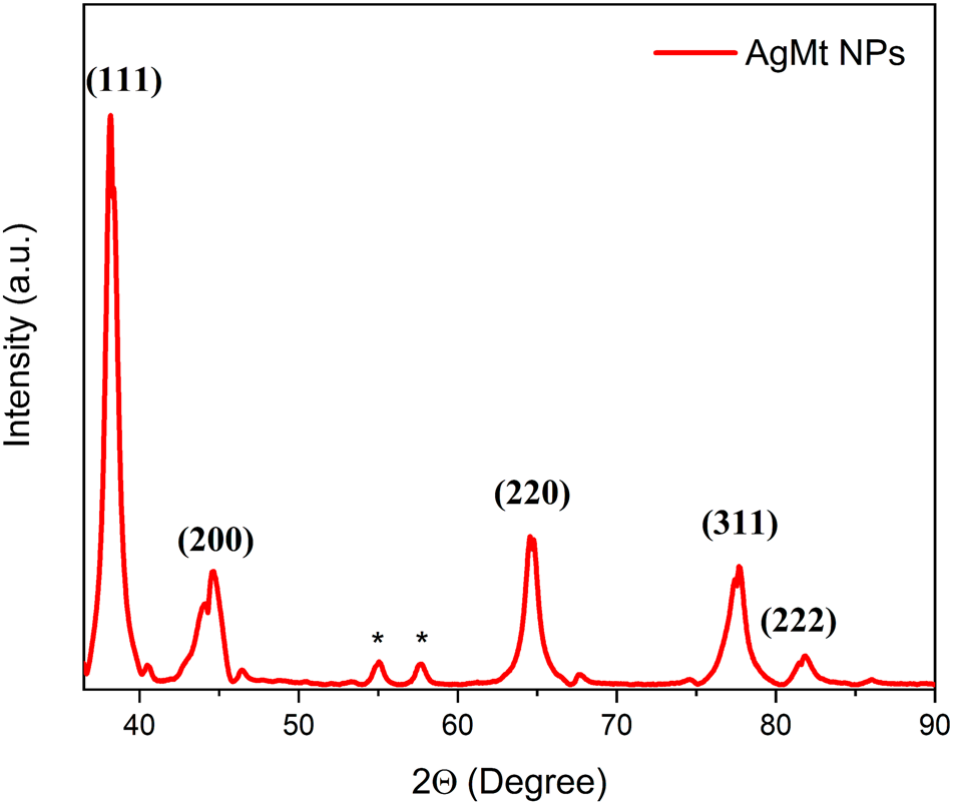
X-Ray Diffraction (XRD) pattern of AgMt Nps powder.

To elucidate AgMt NPs crystalline nature, obtained powders were analyzed by XRD. Figure 3 shows the diffractogram corresponding to AgMt NPs which is characterized by the presence of five intense and narrowed peaks with maxima in $2\theta =$ 38.18 $^\circ$, 44.56 $^\circ$, 64.65 $^\circ$, 77.70 $^\circ$ and 81.85 that could be indexed to (111), (200), (220), (311) and (222) metallic silver fcc (cubic face-centered) planes, respectively, as reported by the Joint Standards Committee of Diffraction (JCPDS File No: 89-3722). AgMt NPs average crystallite size was estimated by Debye–Scherrer formula employing the strongest diffraction peak at 38.18°. The calculated value was 14.95 nm which agrees with the size determined by TEM. Figure 3 also shows some unassigned peaks at 55.05° and 57.66° indicated with (*) which may come from compounds present in the remaining extract that are complexed with AgMt NPs. Precisely these molecules adsorbed on nanoparticles surface provide stability to the system and confer additional therapeutic properties related to these compounds nature^44–46^.

### Zeta potential and DLS

**Figure 4:**
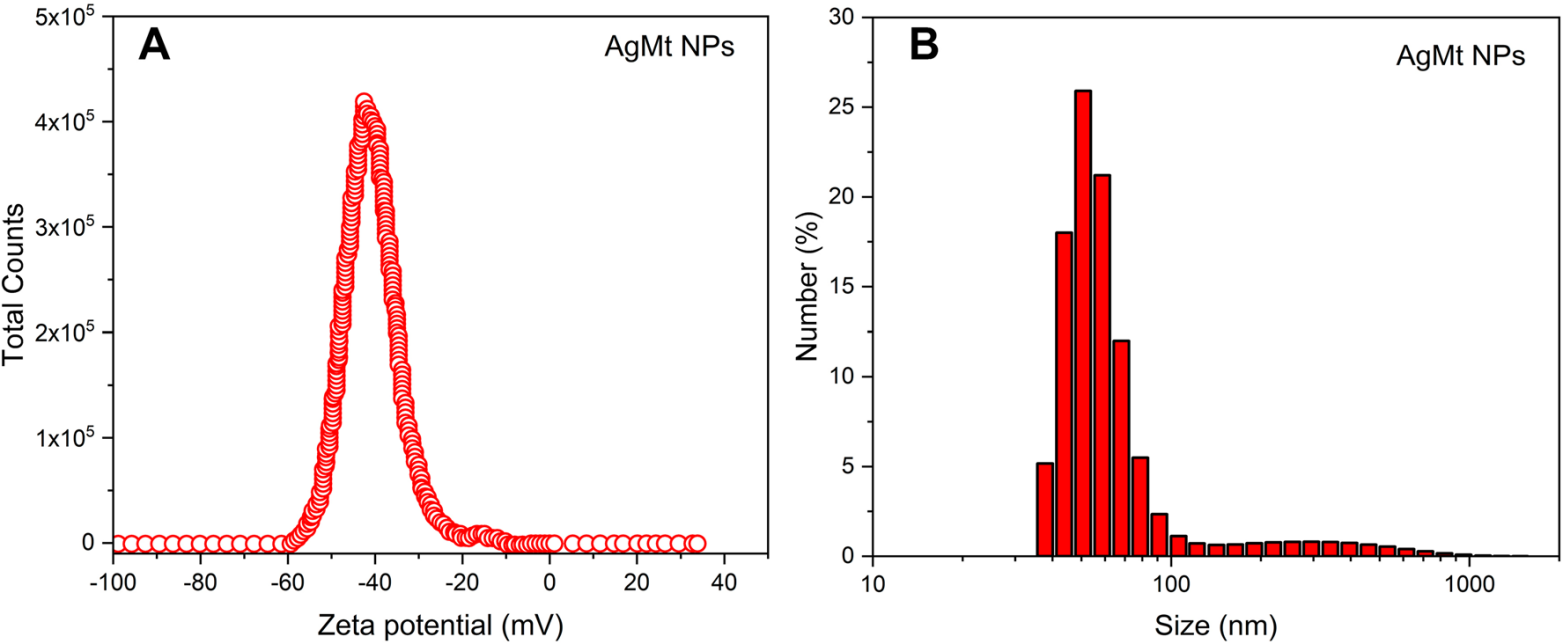
(**A**) Zeta Potential of AgMt NPs and (**B**) Dynamic Light Scattering measurement by number.

Zeta potential is around -41 mV indicated good electrostatic stability for AgMt NPs colloidal dispersion (Fig. 4A). Size distribution by DLS measuring (Fig. 4B) has an average size is 54 nm. As can be seen in the figure, there is also a nanoparticles small population with sizes greater than 100 nm, however, more than 92% of the population have sizes smaller than 100 nm. The difference with respect to size estimation by TEM can be associated with MtE present on nanoparticles surface. For commercial silver nanoparticles (Ag NPs), the Zeta potential is − 24.8 mV (see Supplementary Fig. 2B). Although it is not has a high negative value as AgMt NPs, its value close to − 30 mV indicates acceptable electrostatic stability. Ag NPs dynamic light scattering experiments indicate that commercial nanoparticles have a size mean and size distribution population similar to AgMt NPs (see Supplementary Fig. 2C). These characteristics suggest that selected commercial silver nanoparticles are appropriate to elaborate a hydrogel used as a control in evaluating the bioactive effects of the gels proposed in this work.

### TGA

**Figure 5:**
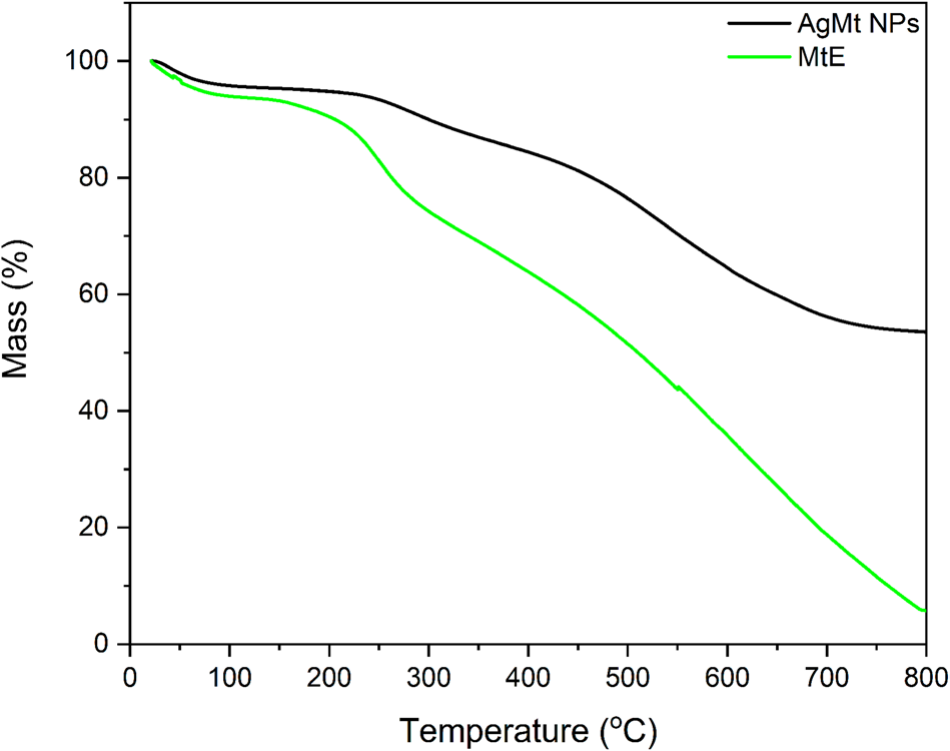
Thermogravimetric analysis (TGA) of MtE, and AgMt NPs.

The organic compounds thermal stability on nanoparticle surfaces was determined using TGA analysis. AgMt NPs final weight at 800 °C was 53.5% of initial value. The weight loss below at 220 °C is around 5% and was attributed to loss water molecules^47^. AgMt NPs weight loss at 800 °C is attributed to degradation of bioorganic compounds present on nanoparticles surface (Fig. 5) whose content was calculated at approximately 46%. This value is similar to that reported by other works of nanoparticles green synthesis^48–50^. AgMt NPs silver concentration determined by atomic absorption is 537.3 µg/mL. From the TGA result, we can estimate MtE concentration retained in AgMt NPs, which is 461.97 µg/mL.

### UV–Vis spectra of gels

**Figure 6:**
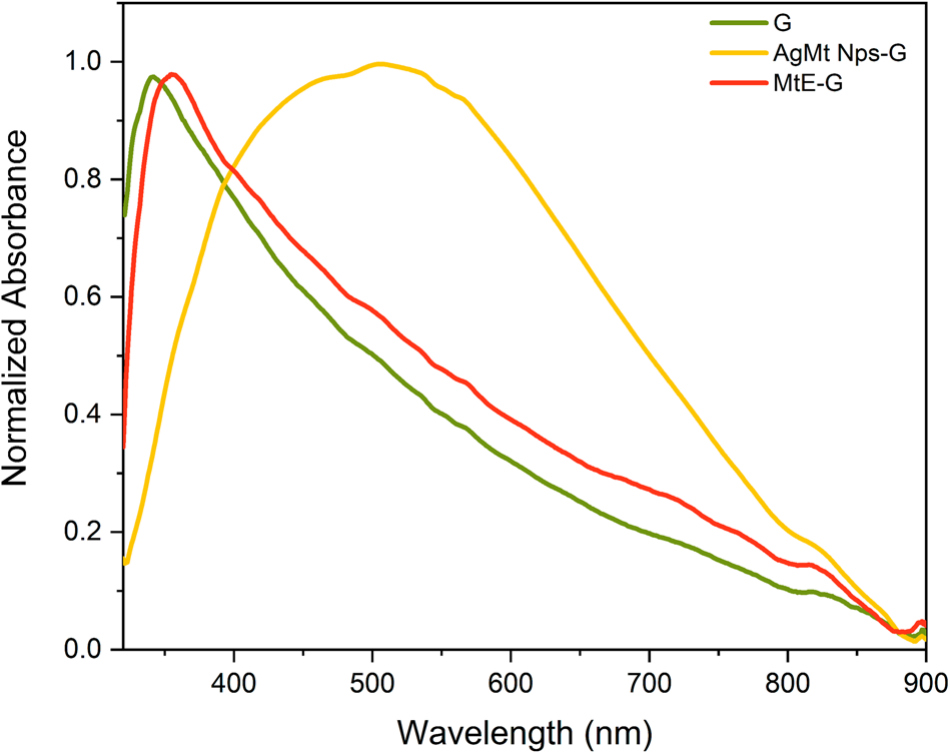
UV–Vis absorption spectra of G, MtE-G and AgMt NPs-G.

In order to identify therapeutic compounds presence of MtE and AgMt NPs dispersed in the support gel, UV–Vis spectra were obtained in the region of 325 to 900 nm as observed in Fig. 6. AgMt NPs resonance plasmon has a maximum at 505 nm and a wider band compared to nanoparticles in aqueous dispersion. This effect can be explained by the presence of the different constituents of the carbopol hydrogel dispersed in the aqueous phase.

### FTIR

**Figure 7:**
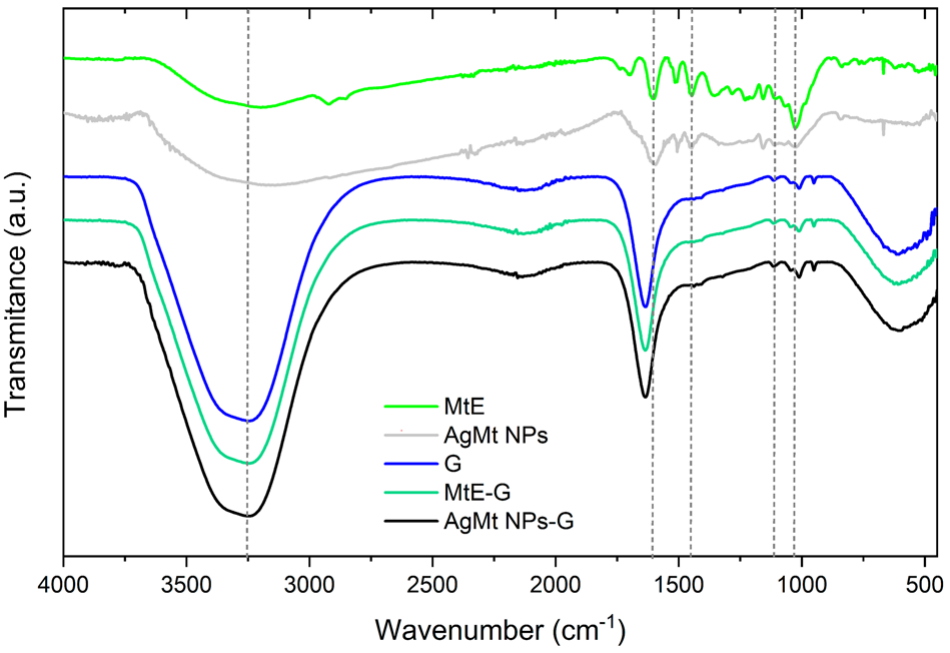
FTIR spectra of MtE, AgNPs, G, MtE-G and AgNPs-G dust dried.

The characteristic broad bands centered around 3250 cm^−1^ are associated with phenolic OH from tannins and flavonoids mainly. Peaks in the range from 1600 to 500 cm^−1^ are identified with polyphenols, signals at 1235 and 1160 cm^−1^ are related with aromatic C–O bond stretching, and at 1020 cm^−1^ to aliphatic C–O band stretching, these peaks in AgMt NPs confirm that NPs (Fig. 7**)** are stabilized by MtE molecules^35,51^. At 1723 cm^−1^ is shifted by oxidation of polyphenolic into carboxylic compounds during the reduction^52–54^ of Ag^+^ to Ag^0^. The peaks at 3248, 1640 the vibrations of carbonyl bonds(C═O), and 620 cm^-1^ are characteristic of the backbone structure of hidrogel for G, MtE-G and AgMt NPs-G. The effect of adding AgMt NPs or MtE to the gel does not produce structural changes^55^. The hydrogel prepared with commercial silver nanoparticles (Ag NPs-G) and used as a control in the bioactivity tests shows the same behavior as the other hydrogels (see Supplementary Figure S3 online).

### Rheometry

**Figure 8:**
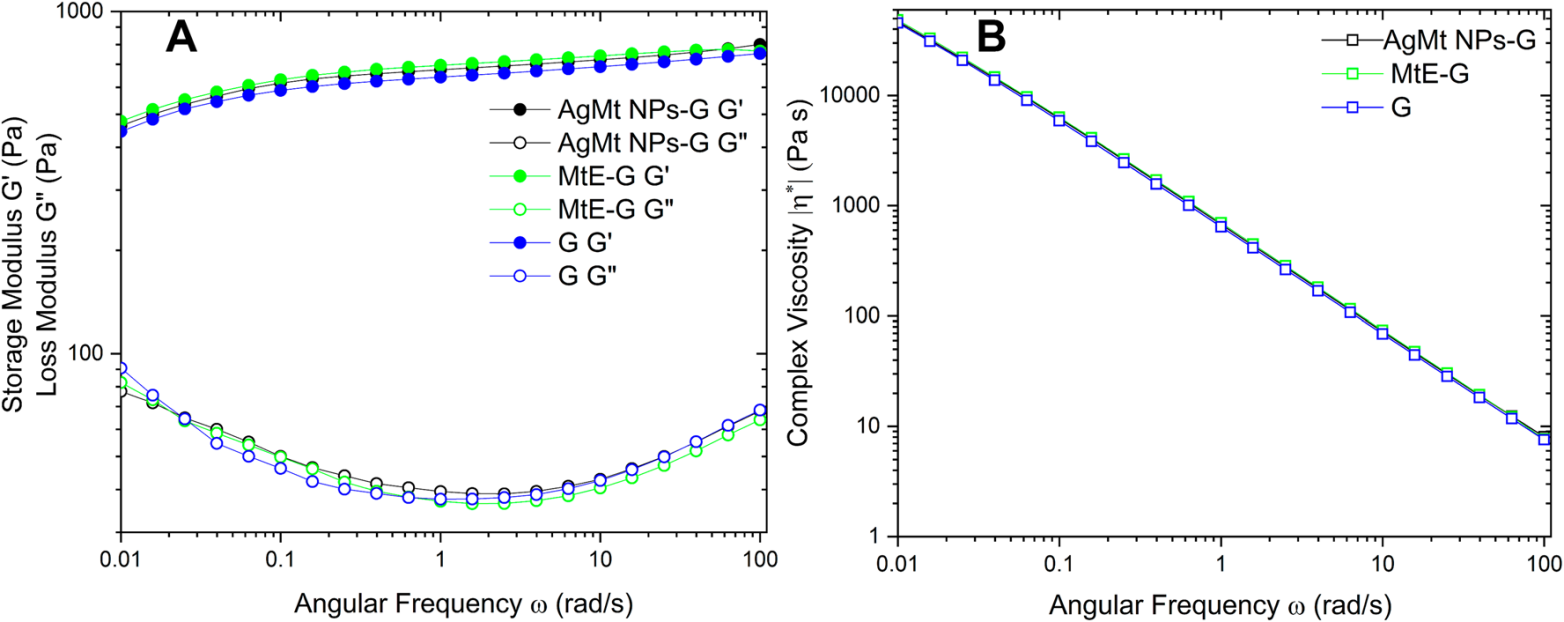
(**A**) Rheometry G’ and G” at 37 o C for G, MtE-G and AgMt NPs-G in angular frequency (rad/s) and (**B**) Complex Viscosity for G, MtE-G and AgMt NPs-G in angular frequency (rad/s).

G^’^ is relationated to the elastic component and the G^”^ with the viscosity, when the storage modulus is higher than loss modulus (G’ > G”) this is indicative of the formation of gel. In Fig. 8A we observe that the effect of adding AgMt NPs (100 µg/g) or MtE to the gel does not produce structural changes in the gel formation (no phase changes are generated)^56^, as observed in the characteristic curves of each system, no modifications are generated in the magnitude of the modules.

Complex viscosity was calculated using the Eq. (2)

2 $$\left| {\eta*} \right| {= }\sqrt {((G')^{2}+(G'')^{2})/\omega}$$

where ƞ^*^ is the complex viscosity and G’ is the storage modulus and G^”^ is the loss modulus^56^.

The negative slope in complex viscosity is feature of non-Newtonian fluid called shear-thinning^57^, in Fig. 8B show how ƞ^*^ decreases with the angular frequency^58,59^.

### Antimicrobial assay

**Figure 9:**
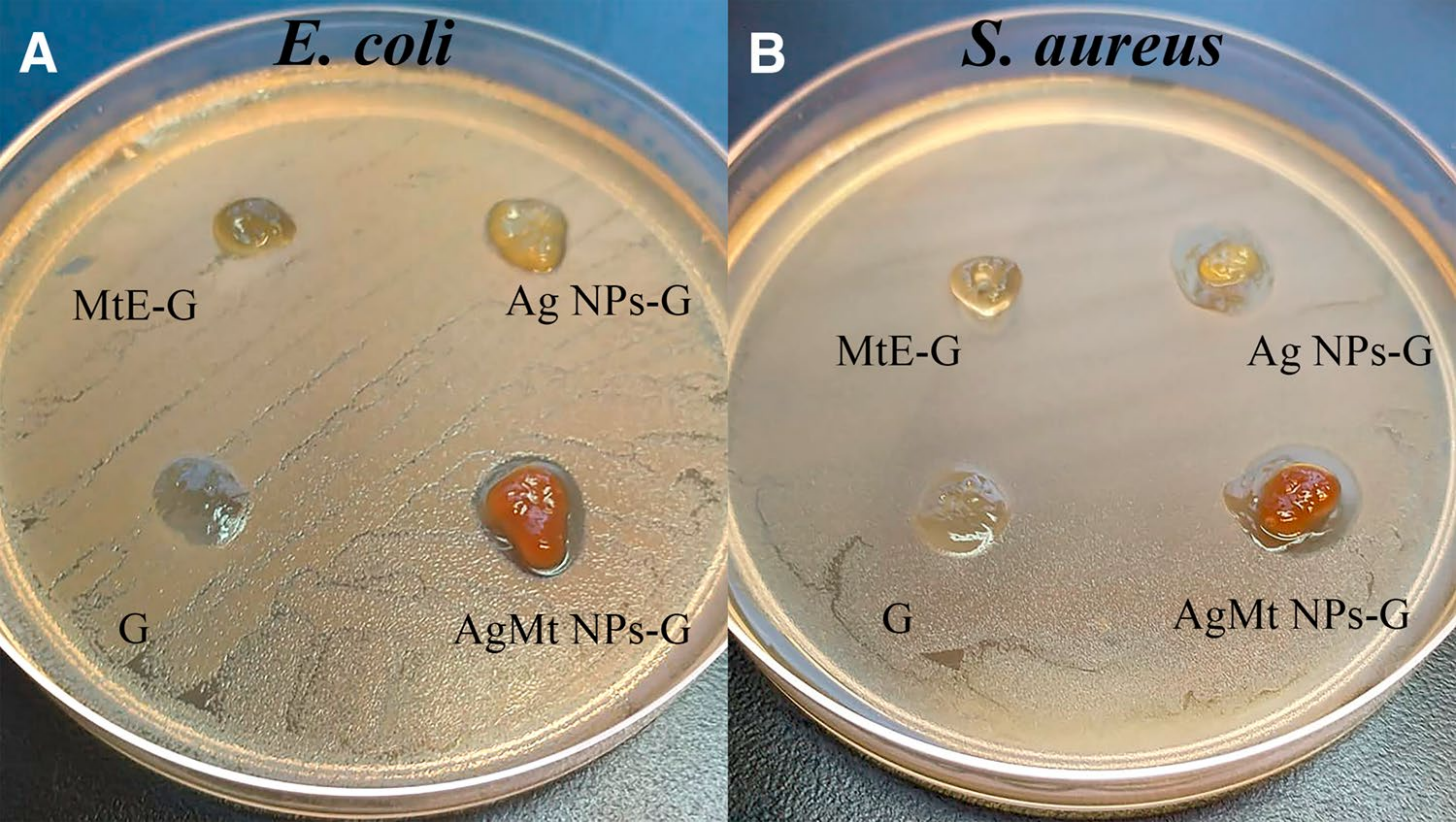
(**A**) *E. coli* (**B**) *S. aureus* Antimicrobial assay.

*E. coli* and *S. aureus* strains were used to evaluate hydrogels microbicidal effects. In Fig. 9A, it is observed that for *E. coli*, AgMt NPs-G produces a considerable inhibition halo while Ag NPs-G generate a much smaller halo. This indicates that growth inhibition is produced by direct interaction between silver nanoparticles and bacteria, where nanoparticles destabilize bacterial membranes, in addition to carrying out numerous interactions with proteins and genetic material, which triggers bacteria death^60–62^. Figure 9B shows that for *S. aureus*, the contact growth inhibitory response is almost the same for both silver gels. This test establishes that of the evaluated materials, AgMt NPs-G produces the best inhibitory response by contact with bacteria, which is desirable in this type of materials applied to superficial wounds in specific regions.

### Minimal inhibitory concentration (MIC) and minimal bactericidal concentration (MBC)

MIC determination was not possible to obtain due to turbidity characteristics of different agents evaluated. In this way all wells were reinoculated in Müeller Hinton agar and MBC was recorded. For cultures in agar, followings results were obtained (Table 1). In this way for *E. coli* MBC for AgMt NPs was 100 µg/mL, for *S. aureus* a very poorly growth was recorded for AgMt NPs, this could indicate that a higher concentration could be a possible MBC value. Also, for *S. aureus* MtE showed an MBC value in 50 µg/mL. For MtE an inhibitory effect on bacterial growth was expected, since in literature it is reported that *M. tenuiflora* extracts possess an important microbicidal activity^63–66^. None one of others (Ag NPs and Vehicle) solutions showed MBC values.

**Table 1:** Minimal bactericidal concentration (MBC) in µg/mL.

	100	50	25	12.5	6.25
***Escherichia coli* ATCC 25922**					
Ag NPs	+	+	+	+	+
AgMt	–	+	+	+	+
MtE	+	+	+	+	+
Ultrapure water	+	+	+	+	+
***Staphylococcus aureus*** **ATCC 6538P**					
Ag NPs	+	+	+	+	+
AgMt	+ ^a^	+	+	+	+
MtE	–	–	+	+	+
Ultrapure water	+	+	+	+	+

+ ^a^ indicate that a very poorly growth was recorded.

### Cytotoxicity

**Figure 10:**
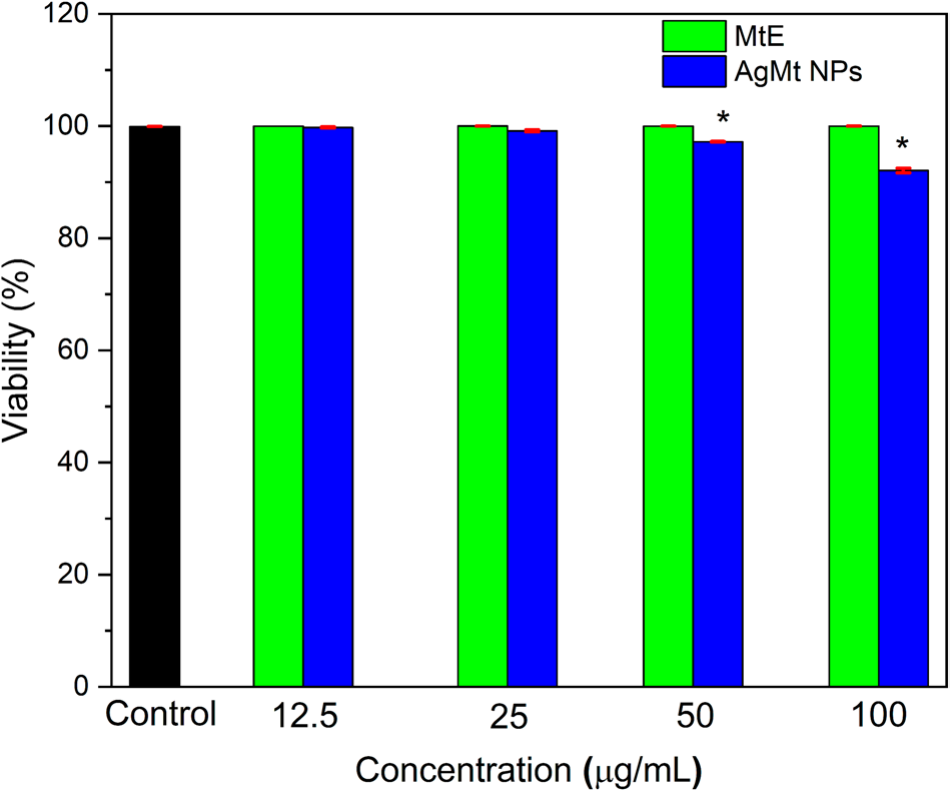
Cytotoxicity results in HUVEC cells. Software Origin(Pro), Version 2018 (https://www.originlab.com/) was employed for statistics ANOVA one-way. The symbol * shows the difference of the means is significant at the 0.05 level for the MtE and AgMt NPs by 50 and 100 µg/mL.

To analyze MtE and AgMt NPs cytotoxic effect HUVEC cells were used. When analyzing the obtained results by FACS using calcein-AM, it is easy to observe that MtE and AgMt NPs tested concentrations for toxicity do not show an important effect on cell viability, except for AgMt NPs 100 µg/mL concentration, where cell viability falls by almost 10% (Fig. 10). There are few works where MtE has been used as a reducing agent for metallic nanoparticles synthesis. In the present work, AgMt NPs toxicity does not exceed 10%, this may be due to several factors, including nanoparticles sizes, metal nature or compounds that are stabilizing the nanoparticles. It is possible that the compounds that reduce metallic precursors for both materials are not the same, therefore, compounds that cover material surface may be playing an important role in nanomaterial biocompatibility. As mentioned previously, there are few published works on nanomaterial synthesis using *M. tenuiflora*, however, *Mimosa pudica* has been used for silver and zinc nanoparticles synthesis. The authors use a gum that is purified from the plant, which contains aromatic compounds, commonly attributed to polyphenols. When evaluating silver nanoparticles toxicity on erythrocytes they observe hemolysis, which is increased as *M. pudica* gum percentage increases for nanoparticles synthesis, where at 10% in concentration of gum a 12% of hemolysis by silver nanoparticles is found^67^.

### Wound healing of second degree burns injuries in Wistar rats model

In the second degree burn model, after 14-day applications, wound healing ratios of AgMt NPs-G (61.22%), were found to be statistically significant (P < 0.05) when compared to control (38.18%), vehicle groups (31.95%) and Ag NPs-G (32.51%) (Table 2). The wound healing ratios of MtE-G (52.15%) were not found to be statistically significant (P > 0.05) when compared to control (38.18%), vehicle (31.95%) and Ag NPs-G (32.51%) groups (Table 2). After 14-day applications, wound healing ratios of AgMt NPs-G were more effective than other treatments.

**Table 2:** Wound healing ratio (% contraction) of second degree burn injuries in wistar rats model.

	Control	Vehicle (G)	Ag NPs-G	MtE-G	AgMt NPs-G
Application	38.18 ± 12.23^a^	31.95 ± 10.69^a^	32.51 ± 11.45^a^	52.15 ± 14.24^a^	61.22 ± 3.26^b^
14-Day	(25.57–50)	(19.92–40.41)	(22.35–38.75)	(36.42–63.24)	(58.34–64.76)

ANOVA analysis was performed (^a^P > 0.05).

ANOVA analysis was performed (^b^P < 0.05).

Measurig the average ± Standar Desviation (SD).

Min–Max value intervarls are in parenthesis.

The wound healing process is divided in four steps such as hemostasis, inflammation, proliferation, and remodeling. The repair process of second degree burn injury needs to coordinate different cellular events involving inflammation, chemotaxis, angiogenesis, repair, and interactions with extracellular components which are necessary for the repair process of wound.

**Figure 11:**
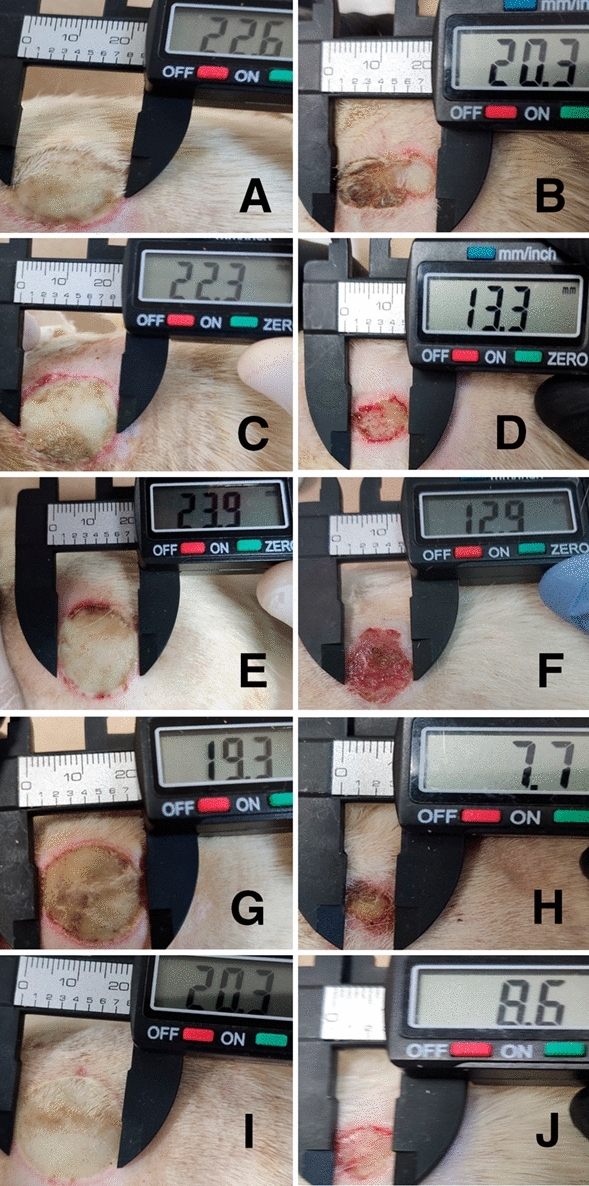
Photograph of the wound healing evolution by the second-degree burn. (**A**) Control group at 0 day. (**B**) Control group at 14 days without treatment. (**C**) Vehicle group at 0 day. (**D**) Vehicle group at 14 days of treatment. (**E**) Commercial silver nanoparticles at 0 day. (**F**) Commercial silver nanoparticles at 14 days. (**G**) MtE group at 0 day. (**H**) MtE group at 14 days of treatment. (**I**) AgMt NPs group at 0 day. (**J**) AgMt NPs group at 14 days of treatment. AgMt NPs group show a faster wound healing evolution compared with other groups.

The macroscopic and histopathological evidence indicate that AgMt NPs-G were more effective than MtE-G, Ag NPs-G, vehicle (G), and control groups in second degree burn healing on Wistar rats for 14 days. Our macroscopical results show that AgMt NPs-G reduce significantly (P < 0.05) the percentage wound contraction when compared with the other groups (P > 0.05). The macroscopic evidence of wound healing evolution is show in the Fig. 11. In this result our observed that AgMt NPs-G group show a faster wound healing evolution compared with other group, this result matches with statistical analyses.

### Histopathological analysis

**Figure 12:**
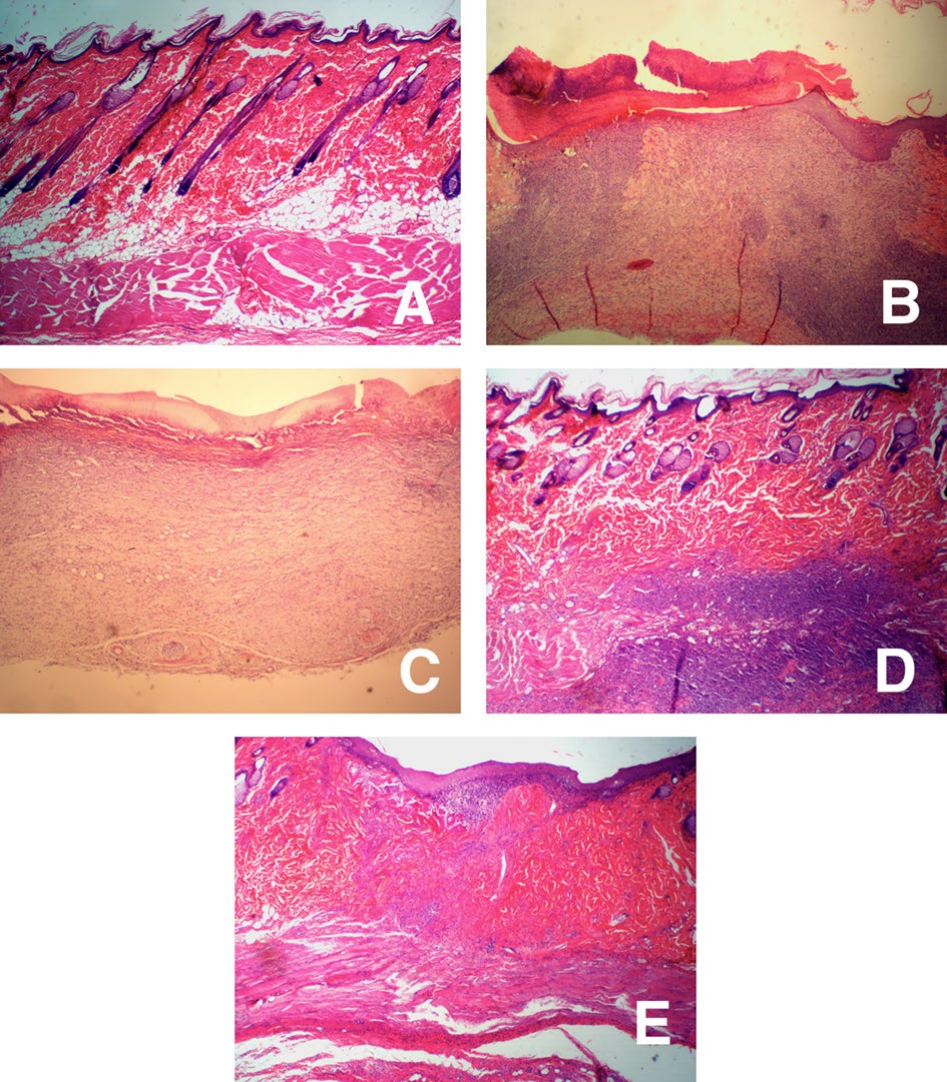
(**A**) Photomicrographs of tissue sections stained with H&E (40X mag), Skin biopsy of the control group showed no histopathological alterations at 14 days. (**B**) Biopsies with the vehicle show a zone of cellular debris, fibrin, hyaline material (crust) as well as a very evident inflammation that extends from the epidermis to the hypodermis. (**C**) Skin biopsies treated with commercial NP, where, instead of the epidermis, an area of necrosis with an inflammatory reaction, edema and newly formed blood vessels is observed, covered by a layer of keratin (presence of scab). At the dermis level, a severe desmoplasia is observed with little diffuse inflammatory reaction composed of lymphocytes, which extends to the hypodermis. (**D**) In the biopsies treated with the Extract, the epidermis and dermis are observed without pathological changes, an evident inflammatory lesion is observed between the deep dermis and hypodermis distributed throughout the entire lesion, presence of sweat glands, hair follicles and the entire epidermis are observed. (**E**) In the treatment with AgMt NPs, are observed basal stratum hyperplasia, moderate acanthosis as well as severe desmoplasia zone, shows integrity of the epidermis, however only some hair follicles were observed.

Control group skin biopsies showed no histopathological alterations in the epidermis, dermis, and hypodermis at 14 days (Fig. 12A). Biopsies obtained from rats treated with the vehicle (G) show a cellular debris zone, fibrin, hyaline material (crust) as well as a very evident inflammation that extends from epidermis to hypodermis (Fig. 12B). Biopsies corresponding to rats treated with the commercial nanoparticles gel (Ag NPs-G) show that instead of the epidermis, an area of necrosis with an inflammatory reaction, edema, and newly formed blood vessels is present, which is covered by a layer of keratin (presence of scab). Severe desmoplasia is observed at the dermis level and a little diffuse inflammatory reaction composed of lymphocytes extending to the hypodermis (Fig. 12C).

In the biopsies obtained from rats treated with MtE-G, the epidermis and dermis are observed without pathological changes, an evident inflammatory lesion is observed between the deep dermis and hypodermis distributed throughout the entire lesion. The presence of sweat glands, hair follicles and the entire epidermis is observed (Fig. 12D).

AgMt NPs-G treatment shows no evident inflammation, epidermis integrity, some hair follicles, basal stratum hyperplasia, acanthosis and severe desmoplasia zone, which is abundant with collagen fibers (Fig. 12E). Several works have reported silver nanoparticles implementation for second degree burn injuries treatment. When the injury is treated with the nanomaterial a rapid tissue repair has been reported, which shows a less inflammation degree compared to untreated or vehicle controls, they also note that there is a greater production of collagen fibers, which generate a greater tensile force^55,68–70^. In the present work, it was also possible to observe the afore mentioned effects, including neovascularization. The observed hyperplasia and desmoplasia may be an adverse effect of the hydrogel matrix compounds, however, it would be necessary to carry out more experiments in order to monitor the progress desmoplasia and hyperplasia.

## Conclusions

In this study, synthesis of silver nanoparticles is reported for the first time using an extract obtained from *Mimosa tenuiflora* bark as a reducing agent. The process is carried out in a single step where the extract molecules act at first instance as reducing agents of Ag^+^ and later as stabilizing agents on synthesized nanoparticles. Characterization techniques indicate that AgMt NPs possess quasi-spherical morphology, with average sizes of 21 nm and fcc crystalline structure, also IR, XPS, and TGA confirm that MtE is present in the AgMt NPs even after the cleaning protocol applied.

Carbopol-based engineered hydrogel provides an efficient matrix to incorporate Ag NPs, MtE, and AgMt NPs. The rheological characterization indicates that hydrogel viscoelastic response is not affected by the incorporation of the therapeutic agents evaluated at a concentration of 100 µg/g providing a stable matrix. Antimicrobial activity tests indicate that AgMt NPs have microbicidal activity on *S. aureus* and *E. coli* with MBC close to 100 µg/mL, and MtE has a microbicidal response on *S.aureus* with MBC of 50 µg/mL. Besides, AgMt NPs-G produces a marked bacterial inhibition by contact in both strains. Histopathological evidence indicates that AgMt NPs-G were more effective than Ag NPs-G, MtE-G, vehicle (G), and control group in second-degree burn healing on Wistar rats for 14 days. The formulation of the hydrogel designed with AgMt NPs is a promising therapeutic agent for burn wound healing with antibacterial and anti-inflammatory effects that enable a more effective recovery in the burn area.

## Supplementary Information

Supplementary Information.
