# Supplementary material for: Hydrogel with silver nanoparticles synthesized by Mimosa tenuiflora for second-degree burns treatment
Source: Sci Rep. 2021 May 28;11:11312. doi: 10.1038/s41598-021-90763-w (PMC8163746; doi:10.1038/s41598-021-90763-w)
Supplement: Supplementary file 1 — Supplementary Information. [file 41598_2021_90763_MOESM1_ESM.docx]

**Hydrogel with silver nanoparticles synthesized by *Mimosa tenuiflora* for**

**second-degree burns treatment**

Aaron Martínez-Higuera^1^, César Rodríguez-Beas^1^, Jesús Mauro Adolfo Villalobos-Noriega^1^, Abraham Arizmendi-Grijalva^1^, Carlos Ochoa-Sánchez^1^, Eduardo Larios-Rodríguez^2^, Juan Manuel Martínez-Soto^3^, Ericka Rodríguez-León^1^, Cristina Ibarra-Zazueta^4^, Roberto Mora-Monroy^5^, Hugo Alejandro Borbón-Nuñez^6^**,** Alfonso García-Galaz^7^**,** María del Carmen Candia-Plata^3^, Luis Fernando López-Soto^3^, Ramón Iñiguez-Palomares^1^*

**^1^** Nanotechnology Graduate Program, Department of Physics, University of Sonora, Rosales and Transversal, 83000 Hermosillo, Sonora, Mexico.

^2^Department of Chemical and Metallurgical Engineering, University of Sonora, Rosales and Transversal, 83000 Hermosillo, Sonora, Mexico

^3^Department of Medicine and Health Science, University of Sonora, Rosales and Transversal, 83000 Hermosillo, Sonora, Mexico

^4^Department of Agriculture and Livestock-University of Sonora. Road to Kino Bay km 20.5, Hermosillo, Sonora, Mexico.

^5^Department of Physic Researching, University of Sonora, Rosales and Transversal, 83000 Hermosillo, Sonora, Mexico

^6^CONACYT-Centro de Nanociencias y Nanotecnología, UNAM, Km 107 Carretera Tijuana-Ensenada s/n, Ensenada, B.C. C.P. 22800, Mexico

^7^Food Science Coordination, Research Center in Food & Development (CIAD), Road Gustavo Enrique Astiazarán Rosas, No. 46, Col. La Victoria, 83304, Hermosillo, Sonora, Mexico

Commercial silver nanoparticles (Ag NPs) purchased from Sigma-Aldrich were characterized by SEM (JSM-7800F, JEOL), and elemental mapping analysis was carried out by energy-dispersive X-ray spectroscopy, EDX (Quantax XFlash-6, Bruker). For this study, 10 µl of a colloidal dispersion of Ag NPs in ultra-pure water (C_Ag Nps_ = 100 µg / ml) was deposited in aluminum foil and dried at 25 ^o^C for 24 hrs before analysis. Other characterizations included DLS, Zeta-sizer, and FTIR.


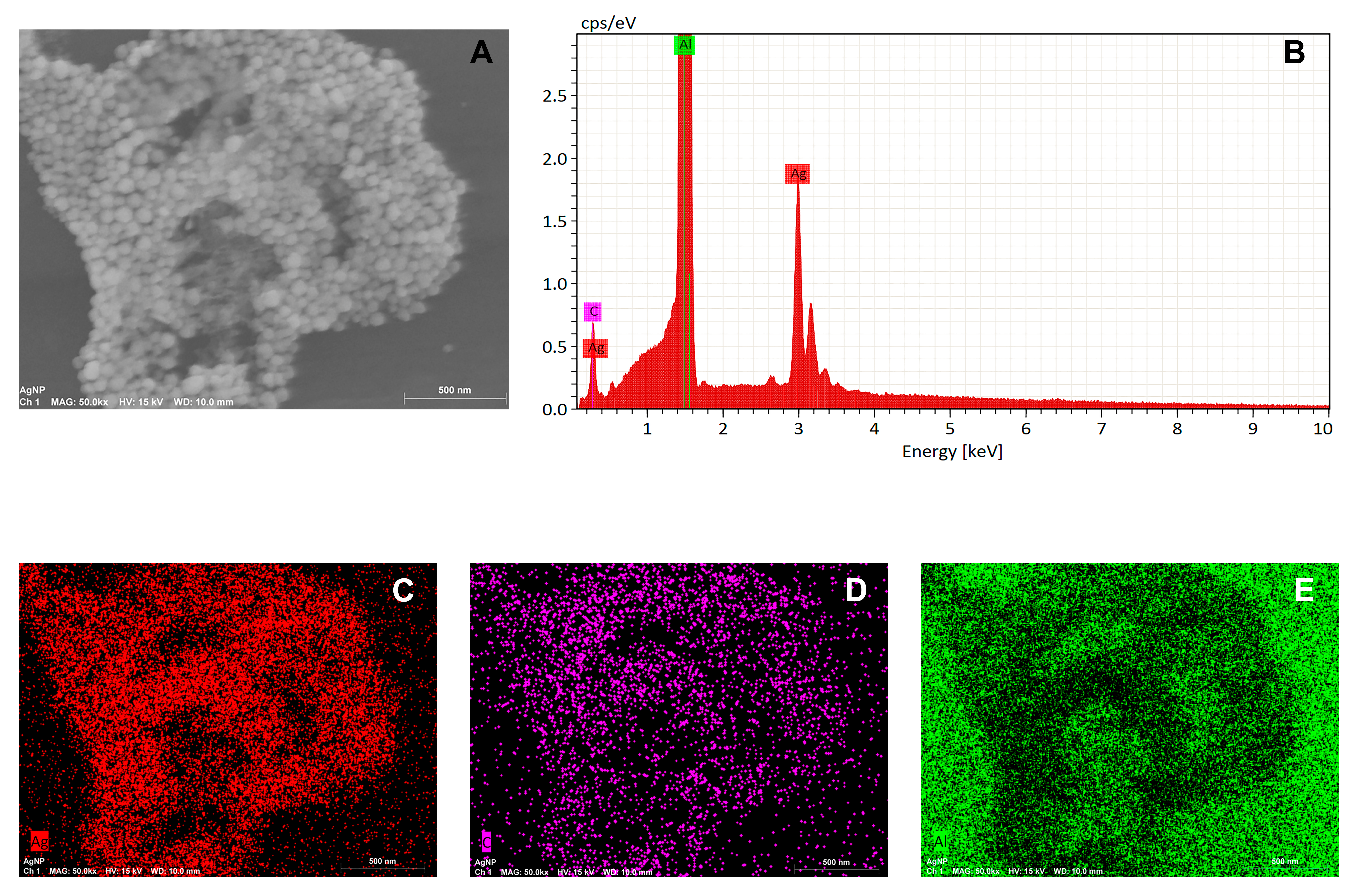


Figure S1: (A) SEM micrograph of an Ag NPs group and their corresponding EDS spectrum (B). Carbon corresponds to polyvinylpyrrolidone (PVP) employed by the manufacturer for nanoparticle stabilization, and aluminum is from the substrate. No oxygen presence indicates that silver oxide is not present in the sample. (C), (D) and (E) correspond to the elemental mapping of Ag, C, and Al, respectively. Carbon merged with silver, indicating that, effectively, PVP stabilizes silver nanoparticles. Aluminum appears distributed in a complementary region of the other elements.


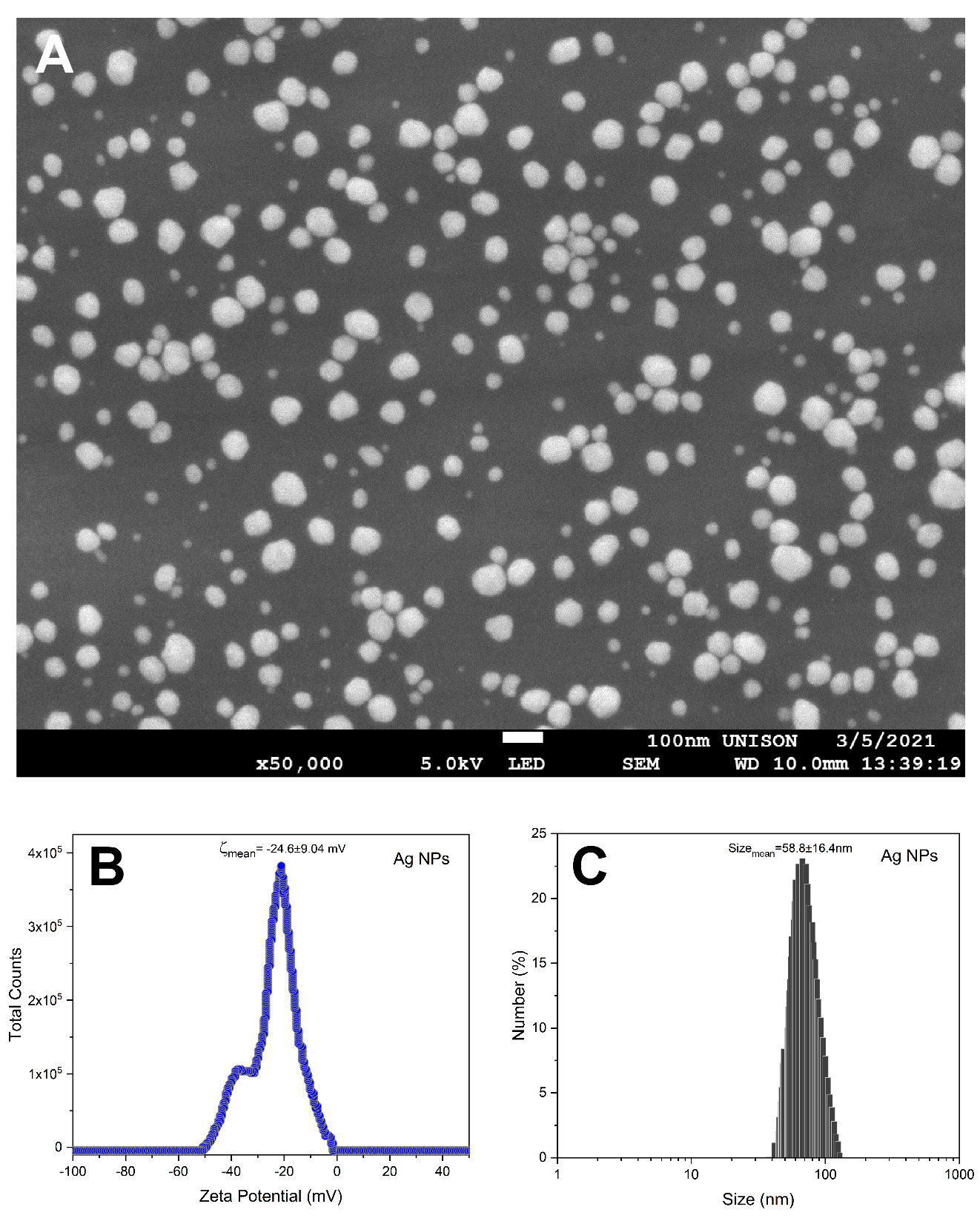


Figure S2. (A) Ag NPs SEM micrograph. Silver nanoparticles are entirely distributed over the surface without agglomeration. (B) The Zeta Potential of commercial silver nanoparticles dispersed in ultrapure water indicates reasonable electrical stability of the colloidal system. (C) Dynamic Light Scattering measurement to size determination by number. Most populations are below 100nm, and the size distribution is close to AgMt NPs system reported in Figure 4B.


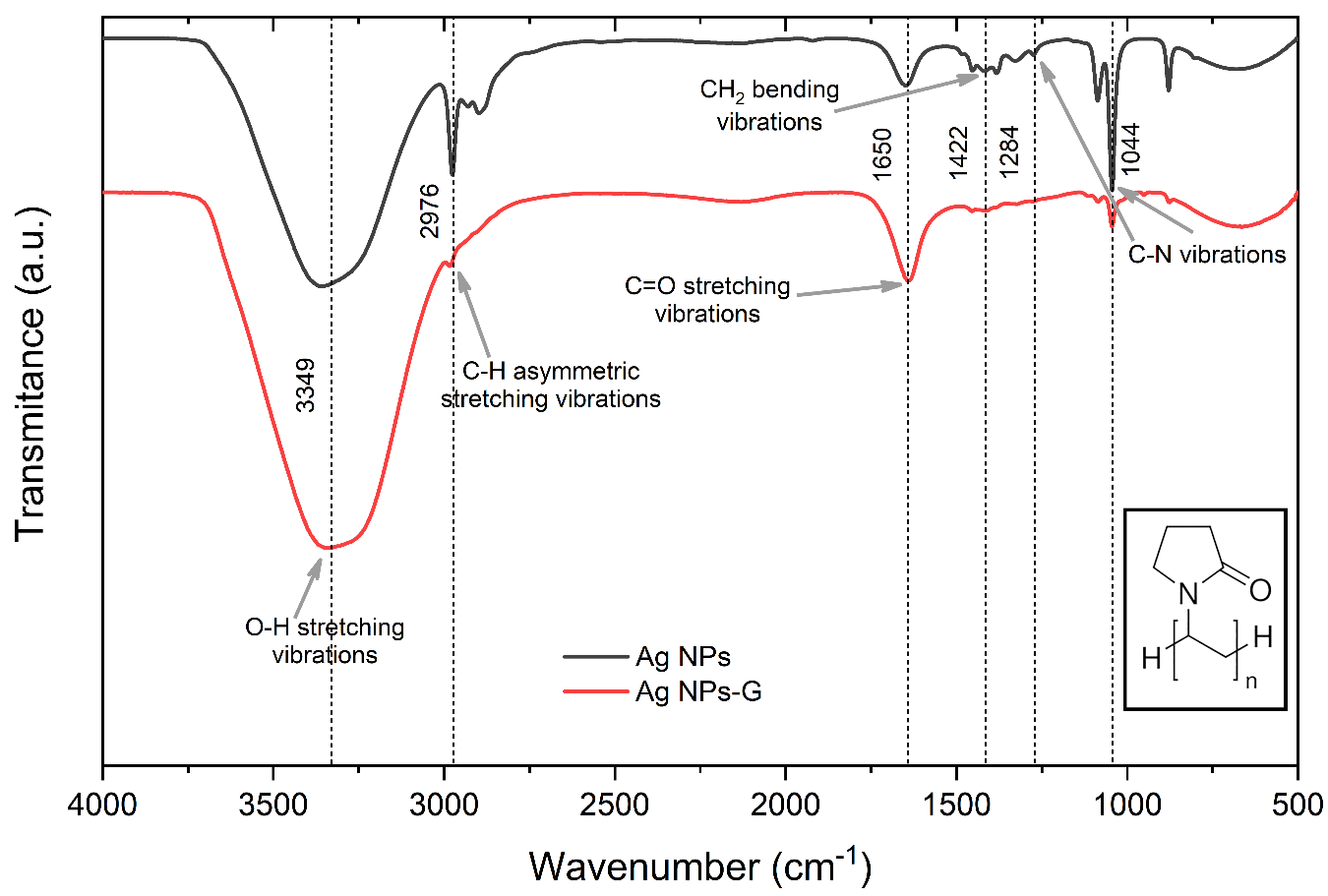


Figure S3. FTIR spectrum of commercial silver nanoparticles (black line) and hydrogel prepared with commercial silver nanoparticles (red line). Signals at 3349, 2976, 1650 cm^-1^ can be assigned to OH, aliphatic CH_2_, and C=O stretching vibrations, respectively. Signals at 1284 and 1044 cm^-1^ are attributed to -CN vibrations from polyvinylpyrrolidone (PVP) present as a stabilizing agent of commercial silver nanoparticles (Ag NPs)^1,2^. On inset, it is showing the molecular structure of the repeating unit of PVP.

**References**

1. Koczkur, K. M., Mourdikoudis, S., Polavarapu, L. & Skrabalak, S. E. Polyvinylpyrrolidone (PVP) in nanoparticle synthesis. *Dalton Trans.* **44**, 17883–17905 (2015).

2. Bryaskova, R., Pencheva, D., Nikolov, S. & Kantardjiev, T. Synthesis and comparative study on the antimicrobial activity of hybrid materials based on silver nanoparticles (AgNps) stabilized by polyvinylpyrrolidone (PVP). *J. Chem. Biol.* **4**, 185–191 (2011).
